# Remodeling the tumor microenvironment to overcome treatment resistance in HPV-negative head and neck cancer

**DOI:** 10.20517/cdr.2022.141

**Published:** 2023-05-30

**Authors:** Sergi Benavente

**Affiliations:** Department of Radiation Oncology, Vall d’Hebron University Hospital Campus, Barcelona 08035, Spain.

**Keywords:** Tumor microenvironments, head and neck cancer, SBRT, immunotherapy, metabolic reprogramming, radiotherapy, HPV-negative

## Abstract

Despite intensive efforts and refined techniques, overall survival in HPV-negative head and neck cancer remains poor. Robust immune priming is required to elicit a strong and durable antitumor immune response in immunologically cold and excluded tumors like HPV-negative head and neck cancer. This review highlights how the tumor microenvironment could be affected by different immune and stromal cell types, weighs the need to integrate metabolic regulation of the tumor microenvironment into cancer treatment strategies and summarizes the emerging clinical applicability of personalized immunotherapeutic strategies in HPV-negative head and neck cancer.

## INTRODUCTION

Head and neck squamous cell carcinomas (HNSCC) develop in close proximity to the anatomical structures responsible for breathing, speaking, chewing, and swallowing. These structures include the pharynx, larynx and oral cavity. HNSCC are common worldwide, with approximately 880,000 new cases and 445,000 deaths (excluding salivary glands) in 2020^[1]^, and are expected to increase by 42% by 2040^[2]^. Up to 60% of patients with HNSCC present with locoregionally advanced disease^[3]^ and usually receive multimodal treatment combining surgery, radiation therapy (RT), and chemotherapy. Even following vigorous treatment, recurrences in the same area or elsewhere have a poor prognosis^[3]^. As a result, there is a great deal of morbidity associated with the treatment for HNSCC, whether it is intended to slow the disease’s course or to cure it entirely. More importantly, HNSCC survivors experience one of the highest rates of suicide likely influenced by the accompanying psychosocial distress and reduced quality of life^[4]^. Multidisciplinarity is a cornerstone of HNSCC management^[5]^ that incorporates diagnostic, therapeutic, prognostic, and patient care collaborative reasoning in the decision-making process. Consensus guidelines are a valuable resource in challenging situations and ensure the quality of tumor board decisions^[6]^. Finally, emergent strategies like the impact of the microbiome in HNSCC can be readily incorporated into multidisciplinary treatment planning^[7]^.

The current treatment strategy is based on tumor location and disease stage, making tumor burden a strong determinant for the treatment decision and outcome, though HPV-positive tumors are associated with a better prognosis^[8]^. Despite recent technological advances aimed at reducing treatment-related toxicities^[9-11]^, local control and overall survival rates in locally advanced disease are rather low, ranging from 40% to 50% at 5 years, especially in HPV-negative cancer^[12]^. Recent developments in immunotherapy have revealed the importance of the tumor microenvironment (TME) in participating in effective immune responses. In recurrent/metastatic HNSCC (R/M-HNSCC), immunotherapy is now a mainstay of treatment, with objective response rates of 13%-17% in an unselected R/M-HNSCC population and 2-year overall survival rates of 17%-27%^[13-15]^, but immunotherapy has failed in the curative setting^[12]^. Equally relevant, genetic and epigenetic events define treatment response. In that regard, it has been shown that HPV-positive tumors with excellent antitumor response harbor a defective DNA repair^[16]^. However, not all HPV-positive tumors hold a better prognosis^[17]^. It is well known that outcomes are poorer for HPV-negative cancers, including those in early stages. In summary, even within histology, tumors may have markedly differing sensitivities.

Hypoxia, which is a hallmark of HNSCC, is a significant impediment to the effectiveness of radiation therapy (RT). Hypoxia, which reduces the induction of DNA double-strand breaks in low oxygen conditions^[18]^ and induces tumor cells to enter a state of quiescence^[19]^, is the cause of radiation resistance. This is essentially the result of aberrant tumor vasculature as well as the high oxygen consumption of a tumor cell population that is expanding at a rapid rate. Both the failure of the treatment and the development of tumor resistance have been connected to hypoxia-induced changes in cellular redox as well as the utilization of alternative metabolic pathways in the TME. Future efforts should focus on differentiating between patients with severely and/or non-correctable hypoxic tumors and those with milder hypoxic characteristics.

Neck nodal disease is common and a well-established adverse prognostic factor in HNSCC^[20]^. Occult metastases in the cervical lymph nodes may be present in patients even if their primary tumors were not particularly large. This has traditionally resulted in the inclusion of elective treatment of the neck as part of the curative therapeutic strategy, being regarded as a factor contributing to survival^[21]^. However, de-escalation strategies suggest that treatment intensity can be modulated. For example, unilateral neck treatment for lateralized oral cavity and oropharyngeal tumors^[22]^ or reduction of RT dose and/or volume to the elective neck in HNSCC^[23,24]^. In the 30 ROC trial, treatment volume and radiation dose were reduced by 60% (i.e., to 30 Gy) in HPV-positive oropharyngeal tumors obtaining excellent results in non-hypoxic tumors^[16]^. Furthermore, recent preclinical models suggest that elective nodal irradiation reduces the efficacy of combined stereotactic radiotherapy and immunotherapy^[25]^. Cumulative evidence suggests that functional lymphatics promote an effective immune response. In contrast, lymphatic remodeling promotes tumor proliferation, indicating that tumor lymphangiogenesis is a causal factor in tumor immune surveillance by the host^[26]^. Preclinical studies have shown that lymph node tumor cells have the ability to reach other organs via high endothelial venules^[27,28]^, which is an intriguing fact to take into consideration. As a result, the development of novel treatments is made possible thanks to a deeper comprehension of the dynamic relationship that exists between lymphatics, tumor cells, and the TME.

This review explores how immune and stromal cells in the TME influence immunosuppression and proposes targeting strategies to promote immunomodulation, weighs the integration of metabolic regulation of the TME into treatment strategies, and summarizes the emerging clinical applicability of personalized immune therapies in HPV-negative HNSCC.

## KNOWLEDGE GAPS IN THE CURRENT THERAPEUTIC LANDSCAPE IN HPV-NEGATIVE HNSCC

A high tumor mutational burden is characteristic of HNSCC, and this has been identified as a critical factor in immune response in other malignancies. Furthermore, HNSCC tends to exhibit tumor-infiltrating immune cells, which are a recognized prognostic factor in a subset of HNSCC subtypes. However, the impact of immunotherapy on HNSCC has been rather low, with HNSCC falling into certain subtypes and that have a high level of resistance^[29,30]^. Reevaluating the treatment for these tumors, which are not immunogenic, is one way to improve the standard of care for these cancers.

### Prognosis

The stage and HPV status are recognized as the major determinants of HNSCC prognosis. However, it is becoming clear that not all HPV-positive HNSCC share the same good prognosis^[17]^ and that not all HPV-negative HNSCC are the same^[31]^.

Anatomical location, patterns of lymphatic drainage and patient preference, in combination with technical/technological requirements/skills and foreseen toxicities delineate curative treatment strategies in HNSCC. Nevertheless, hypoxia and the TME strongly affect the efficacy of immunotherapy^[32]^. An understanding of the relative variations in the different anatomical sites of HNSCC might assist in the planning of new and more effective treatment strategies. A recent evaluation of the TCGA dataset concludes that HPV-negative sites are molecularly different, especially between tumors of the oral cavity and larynx^[31]^. In this study, larynx cancer had a higher mutational burden, and was enriched for neuronal and glycosylation pathways, with a greater abundance of B cells and endothelial cells; while oral cavity cancer was enriched in extracellular matrix (ECM) pathways, with a greater abundance of monocytes and greater methylation of Hox genes; oropharyngeal cancer was the most hypoxic, and oral tongue cancer had a higher abundance of dendritic cells (DCs).

The immune system’s decreased recognition of the tumor or inhibition of its response may explain why some HNSCC respond to immunotherapy, but many others behave as resistant and are regarded as poor immunogens (see excellent review in ref.^[33]^).

### Landscape of the immunosuppressive TME in HPV-negative HNSCC

**Figure 1:**
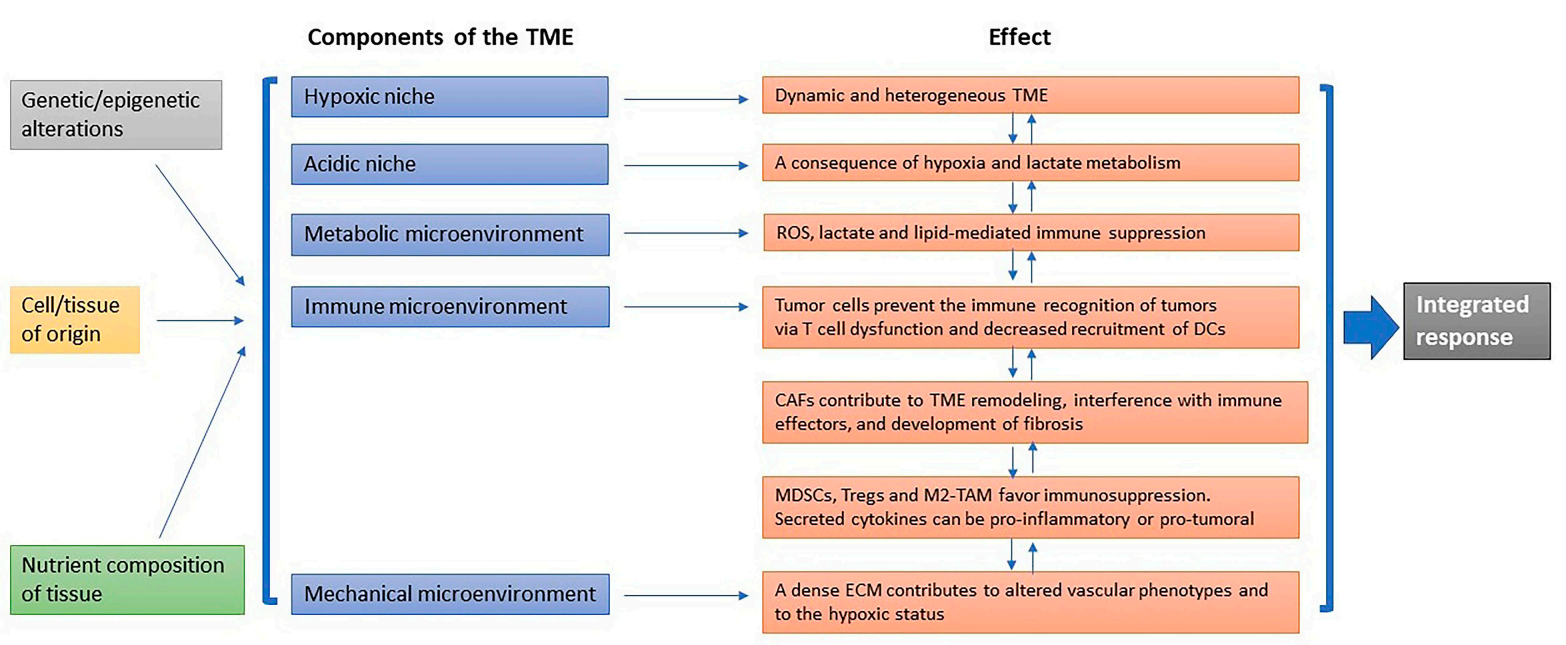
Dynamic composition of the TME. Specialized microenvironments that can be influenced by the genetic/epigenetic background, the tissue of origin, and the components of the tissue, and promote specific effects that are dynamically interconnected. The final outcome results from an integrated response.

The TME is heterogeneous and can be envisioned as a core of specialized microenvironments disposed as intersecting paths that can reprogram cancer biology and serve as potential targets of cancer therapy [Figure 1].

#### Spatial architecture of the tumor versus immune evasion

A global hypoxic state is facilitated by a dense ECM, which limits immune cell infiltration and prevents their antitumor effect. Increased angiogenesis, the recruitment of additional immunosuppressive cells, tumor progression and enhanced metastatic ability are all factors that contribute to a global hypoxic state. Tissue microarrays of human HNSCC confirmed the vascular heterogeneity observed in patient‐derived xenograft (PDX) models of HNSCC. These models showed three distinct vascular phenotypes, including a tumor vessel (TV) phenotype in which the majority of the blood vessels were distributed throughout the tumor; a stromal vessel (SV) phenotype in which the majority of the vessels were restricted to the infiltrating stroma next to the tumor cells; and a mixed vessel phenotype. In HPV-negative HNSCC, a heterogeneous distribution of vascular phenotypes was seen, with 60% of the tumors belonging to the SV phenotype, 25% of the tumors belonging to the mixed vessel phenotype, and 15% of the tumors belonging to the TV phenotype. A better response to antivascular agents was seen in the TV phenotype, but not in the SV phenotype^[34]^. Understanding the physical barriers involved in HNSCC opens up the possibility for localized drug delivery, which could limit the systemic effects of different immunotherapies.

#### Tumor cells versus immune recognition of tumors

Conditions that prevent the proper priming, activation and infiltration of T cells are known to compromise the immune response. Thus, the T cells that are present in poorly immunogenic tumors are dysfunctional, exhausted or excluded from the TME. T cell exhaustion (TEX) is considered a major limitation to the long-term efficacy of immunotherapy. The complex immunosuppressive network in the TME facilitates the majority of CD8^+^ T cells evolving into the exhausted T cell subtype^[35]^. TEX is not a static process with only one conceivable end; rather, it comprises a large variety of transitional phases. Instead, the pool of exhausted CD8^+^ T cells that are found in the TME should be considered as a population of CD8^+^ T cell subsets that have varying degrees of TEX, and each has its own unique set of functional capabilities ^[36]^. In this regard, a pan-cancer analysis of the heterogeneous TEX subset landscape using a five-stage trajectory as measured using TEX-specific hierarchical developmental signaling pathway signatures reveals the interdependencies of the TEX subgroups in various types of tumors, as well as among the same types of tumors. TEX patterns in HNSCC TCGA molecular subgroups revealed that the highly immunogenic progenitor TEX (TEX^prog^) subset was particularly enriched in the HNSCC basal type, while the three remaining molecular types (atypical, mesenchymal and classical) demonstrated intermediate distribution of transcriptional factors T cell factor 1 (TCF1, marks a downstream population of stem-like precursors of CD8^+^ T cells characterized by high self-renewal capacity, proliferation, and polyfunctionality^[37]^), T-box expressed in T cells (T-bet; involved in effector, memory and exhausted CD8^+^ T cell differentiation^[38]^), and thymocyte selection-associated high mobility group box (TOX, essential role in the induction of TEX by mediating transcriptional and epigenetic changes) known to coordinate the dynamics underlying TEX subset transitions^[39]^.

Decreased recruitment of DCs, required for tumor antigen presentation, can also contribute to immune escape by preventing adequate T cell priming and trafficking into the TME. Both classical DC (cDC1, cDC2) and plasmacytoid DC (pDC) are distinct subsets of the DC compartment. Batf3-driven CD8α^+^/CD103^+^ cDC1 are thought to be the major mediators of antigen transport and cross-priming since they are the most common cross-presenting DC subgroup. While cDC2/moDC and pDC have been shown to have stimulatory roles in some contexts, these cells’ roles are often more tolerant in the TME ^[40]^. Tissue-specific DCs and tumor-associated macrophages (TAMs) can be generated in the environment of the tumor, inhibiting the anticancer immune response. The relevance of tissue-derived APCs to anticancer immune responses is highlighted by the plasticity of the myeloid compartment in response to the microenvironment. Learning how tissue-specific APCs differ in their function will aid in the discovery of cutting-edge cancer immunotherapies^[41]^.

It is common knowledge that various other cell types are also capable of carrying out the process of exogenous antigen presentation. These cell types are grouped together and referred to as “amateur” APCs^[42]^. There is accumulating evidence to suggest that the expression of HLA class II antigens on tumor cells has a major impact on immunogenicity^[43]^. There is a correlation between favorable outcomes and high levels of constitutive HLA class II antigen expression in oropharynx cancers^[44]^. It is possible that the favorable effect of HLA class II antigen expression is due to the presence of high amounts of interferon gamma (IFNγ) in tumors that have substantial infiltration by T cells.

#### Immunosuppressive cells in the TME vs immune effectors

Cancer-associated fibroblasts

Cancer-associated fibroblasts (CAFs) participate very actively in the TME remodeling and interfere with immune cell effectors, rendering them ineffective. High CAF density is correlated with disease stage and poor prognosis in HNSCC^[33]^. CAF-induced recruitment of regulatory T cells (Tregs) through transforming growth factor beta (TGF-β) and interleukin 6 (IL-6) inhibits the proliferation of CD8^+^ T cells in HNSCC^[45]^.

CAFs-TME remodeling. CAFs are the most common type of cell to be seen in the stroma of a tumor. These CAFs secrete cytokines, which can either act on many immune cells at the same time or affect the TME in some other way to influence immune cell infiltration. CAFs are responsible for the release of soluble molecules, such as IL-6, which have an effect on T cells, NK cells, DCs, TAMs, and neutrophils. Single-cell RNA sequencing has previously been used to identify three distinct CAF types in HNSCC; these types include myCAF and two undefined CAF subtypes (CAF1 and CAF2). Despite this, the functional significance of these subtypes and their relationship to the immunotherapy response are still unknown^[46]^. A study that used protein activity patterns, as determined by the VIPER algorithm analysis of a longitudinal single-cell transcriptomics HNSCC dataset^[47]^, indicated that there are five CAF subtypes that are distinct from one another on a molecular level. The HNCAF-0 and HNCAF-3 subtypes were predictive of favorable clinical responses to PD-1 immune checkpoint blockade (ICB), and they were connected to improved CD8^+^ T cell cytotoxicity (pro-inflammatory CAFs). Tissue-resident memory (Trm) phenotype CD8^+^ T cells were created by co-culturing HNCAF-0/3 with CD8^+^ T cells. These cells co-expressed CD94/NK group 2 member A (NKG2A, CD159), an inhibitory receptor that is substantially abundant in tumor-infiltrating Trm^+^ CD8^+^ T cells in HNSCC^[48]^. As activating NKG2A with its ligand HLA-E reduces cytotoxicity and effector function, this protein has the potential to be used as a new target for immunotherapy^[49]^. In clinical trials involving HNSCC, the use of a combination of NKG2A inhibition and other checkpoint inhibitors has been shown to have a positive effect on the patients^[50]^. Additional evidence has been reported in bladder cancer, suggesting that bladder tumors having both high levels of HLA-E and NKG2A-positive CD8^+^ T cells could benefit the most^[49]^, and in radioresistant tumors that do not respond to combined RT and ICB^[51]^. High-resolution single-cell sequencing CAF analyses constitute an excellent framework to develop strategies to reprogram CAFs towards the pro-inflammatory phenotype, identify novel combinations with immunotherapy, and the potential to establish HNCAF subtypes as biomarkers of response and resistance in future clinical trials^[47]^.

The proliferation of CAFs is a critical step in the development of fibrosis in the tumor stroma^[52]^. Multiple studies have pinpointed a critical function for the anti-apoptotic protein Bcl-2-associated athanogene 3 (BAG3) in tumor cell signaling in the TME^[53]^ and in the progression of fibrosis in tumor tissues^[54]^. BAG3 causes TAM activation and IL-6 production in pancreatic tumor cells. The close link of BAG3 expression with cancer fibrotic phenotypes^[55]^ suggests that anti-BAG3 therapy dramatically down-modulated the expression of a-SMA, an activation hallmark of CAFs, with a marked reduction of collagen buildup. HNSCC has been identified as a fibrotic tumor phenotype that is more likely to respond to anti-BAG3 therapy, as determined by an analysis of three distinct databases including high-throughput RNA sequencing information from PDXs^[56]^.

Myeloid-derived suppressor cells

Myeloid-derived suppressor cells (MDSC) trafficking-metabolism. Monocytic (M-MDSC), granulocytic polymorphonuclear (PMN-MDSC), and early stage (e-MDSC) are the three subsets of MDSCs recognized; e-MDSCs lack the myeloid lineage markers found in the first two groups^[57]^. It has been found that both PMN-MDSCs and M-MDSCs are linked to T cell suppression^[58]^. The presence of PMN-MDSCs also in the bloodstream ^[59]^ has been linked to poor survival^[60]^ and TEX^[61]^.

It has been observed repeatedly that RT induces MDSC expansion coupled to PD-L1 upregulation on the surface of MDSCs. This appears to occur approximately 2 weeks after RT and correlates with high levels of IL-6 and arginase activity^[62,63]^. In addition, the production of reactive oxygen species (ROS) is caused by RT, which dramatically activates the hypoxia-inducible factor 1 subunit alpha (HIF1α) in cancer cells^[64]^. As HIF1α signaling in malignant cells has been consistently associated with MDSC accumulation in the TME and enhanced immunosuppression^[65]^, and ROS control MDSC functions^[66,67]^, research is required to translate targeting strategies of MDSC-dependent immunosuppression into appropriate clinical scenarios, such as in patients with marked MDSC expansion/activity^[68]^.

Regulatory T cells

**Figure 2:**
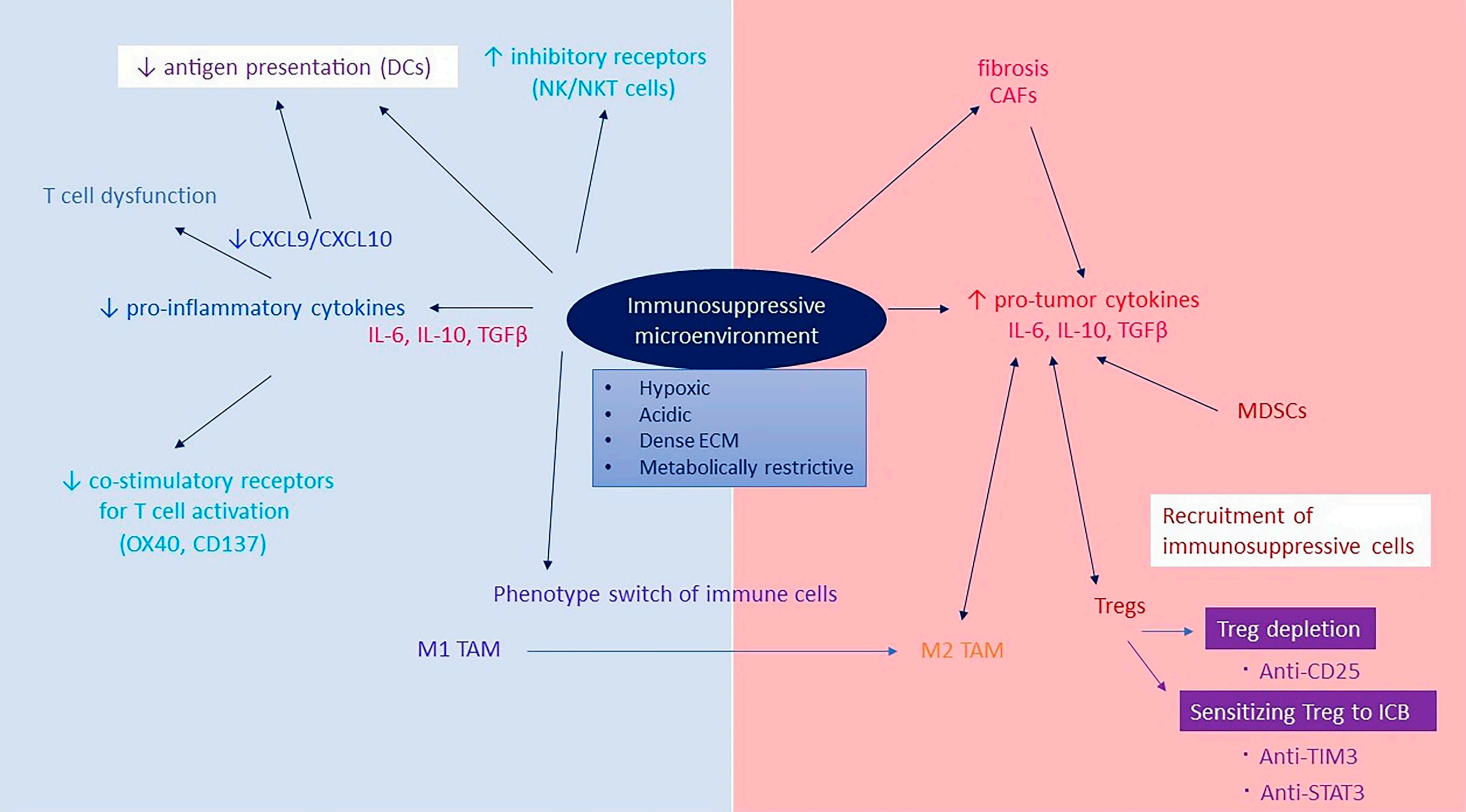
Immunosuppressive tumor microenvironment. Direct and indirect alterations in the TME suppress the immune response. Pro-tumor cytokines recruit immunosuppressive cells that prevent antigen presentation, which could be addressed by targeting Tregs. Reduction of pro-inflammatory cytokines generates T cell dysfunction and reduced antigen presentation. CAFs: cancer-associated fibroblasts; DCs: dendritic cells; ICB: immune checkpoint blockade; IL-6: interleukin 6; IL-10: interleukin 10; MDSC: myeloid-derived suppressor cells; TAM: tumor-associated macrophage; TGF-β: transforming growth factor beta; TME: tumor microenvironment.

Regulatory T cells (Tregs) constitute a unique subpopulation of CD4^+^ T cells characterized by expression of the forkhead box P3 (FOXP3) transcription factor and high levels of CD25, a component of the IL-2 receptor. Tregs play a major role in restraining tumor-associated antigen-specific immune responses. It has been demonstrated that some markers, such as CTLA4, TIM3 and STAT3, play a part in the process of mediating resistance to RT and ICB. The suppressive effects of several immune populations, including Tregs, are known to be increased by these markers ^[69]^. It seems that activated Tregs can suppress effector cells with inhibitory cytokines, metabolic competition, or direct inhibitory action on effector T cells (Teff) and DCs by secreting inhibitory cytokines, engaging in metabolic competition or taking direct inhibitory action [Figure 2]. Such findings have been documented in HNSCC patients treated with cisplatin-based chemoradiation, where > 20% elevations in circulating MDSCs were detected and correlated with an increase in Tregs and with suppressed T cell responses^[70]^.

Treg-APC axis. In immunologically cold HNSCC, characterized by limited T cell infiltration and poor responsiveness to RT plus anti-PD-L1 combination, antitumor immunity produced by RT can be improved through regulation of the APC-Treg axis. Treg depletion alone appears to be insufficient in tumors poorly infiltrated by Teff, which likely explains that Treg depletion combined with immunotherapy will not work. Conversely, RT is an excellent strategy to transform poorly immunogenic tumors owing to its immune-boosting properties^[71]^. RT plus anti-CD25 seems to be effective only in tumor models unable to induce infiltration of MDSCs, suggesting that multiple immune-suppressive populations may need to be targeted to induce a robust antitumor immune response by RT^[72]^. Preclinical HNSCC models have shown that treatment with anti-TIM3 plus anti-PD-L1 combined with RT^[73]^ and anti-STAT3 plus RT^[72]^ are potent therapeutic strategies against Tregs in appropriate tumor models. The activation of DCs and stimulation of their ability to mature and move to the lymph node have been important for eliciting a Teff response in this highly resistant HNSCC, even though this combination can effectively eliminate several tumors in animal models. In preclinical models, tumor eradication occurs when RT is coupled with anti-CD25 (Treg depletion) and anti-CD137 (DC agonism) treatment^[74]^. Previous studies have demonstrated that tumor necrosis factor receptor superfamily member 9 (CD137,4-1BB) can enhance DCs and reprogram Tregs^[75]^. Reprogramming Tregs into Foxp3^+^ CD4^+^ T cells with cytolytic activity was significantly aided by modulating the interaction of CD137 with its ligand. To emphasize the significance of increased antigen release for tumor eradication, it is important to note that these results were only accomplished with hypofractionated RT^[74]^.

#### Cytokine responses *vs.* immunometabolic reprogramming

Cytokines are small molecular messengers that affect immune cell proliferation, differentiation and activation, hence regulating lymphoid tissue development, immunity and inflammatory responses by controlling immune cell growth, differentiation and activation. The HNSCC TMEs are abundant in immunosuppressive phenotypes (Tregs, TAMs, MDSCs, pDCs, CAFs) which cause immune effector cells to be either excluded from the tumor or to become dysfunctional. These stromal cells not only supply the tumor cells with intermediary metabolites and nutrients, but also produce a large quantity of pro-inflammatory and proangiogenic cytokines, which together create a pro-tumorigenic environment and aid in the evasion of the antitumor immune response (see review in ref.^[76]^).

It is possible for tumor cells to directly secrete immunosuppressive and anti-inflammatory cytokines, such as interleukin 10 (IL-10) and TGF-β, which negatively affect APCs and T cells; other cytokines can polarize immune effector cells toward adopting an anti-inflammatory phenotype that leads to tumor progression. Expression of anti-inflammatory cytokines (IL-10, IL-6, and TGF-β) is favored in HPV-negative HNSCC. Mediating communication between tumor cells and CAFs^[77]^ and playing a crucial role in hypoxia-induced MDSC accumulation, elevated serum IL-6 levels are linked to increased tumor burden and aggressiveness^[78]^. IL-10 affects the functional capacity of tumor-infiltrating pDCs, promoting the expansion of Tregs^[79]^, and is a potential predictor of a poor clinical outcome for the treatment of HNSCCs of laryngeal origin^[80]^. TGF-β suppresses Teff cells and promotes Tregs^[81]^. Recent research suggests that a myeloid PD-L1 blockade combined with TGF-β depletion in HPV-negative HNSCC can increase CD8^+^ T cell infiltration in responders showing a more permissive TME for Teff function^[82,83]^. Thus, TME cytokine milieu-targeted therapies can either increase the production of pro-inflammatory cytokines or decrease the production of pro-tumor cytokines. More research is needed to establish the best way to manipulate local cytokines in order to modify the TME, especially in conjunction with other medications.

Chemokines are chemotactic cytokines capable of moving receptor-expressing cells along chemical gradients. The role of chemokines in cancer is conflicting in that they may facilitate the migration of tumor cells as well as attract tumor-infiltrating immune cells^[84]^. Patterns of chemokine/ligand-receptor expression are emerging. Growing data indicate that the CXCR3 ligands CXCL9 and CXCL10, induced by IFNγ, play an important role in immune-inflamed tumors. In contrast, even if CXCL9 and CXCL10 are present in immune-excluded tumors, the spatial exclusion of Teff cells may represent the abundance of MDSCs, which stimulate the formation of a dense stroma that restricts the T cell entry^[85]^.

Studies of chromosome somatic copy-number alteration (SCNA, or aneuploidy) profiles have mapped the chromosomic alterations driving tumors to show reduced expression of cytotoxic infiltrating immune cells that predict response to ICB, being the strongest signals for HNSCC and pancreatic cancer^[86]^. In HPV-negative HNSCC, 9p21.3 loss was associated with depletion of cytotoxic T cell infiltration in *TP53* mutant tumors; and in oral cancer, 9p-arm level loss was the strongest driver of cytotoxic T cell depletion (mainly of CD8^+^ T cells), promoting profound decreases of IFNγ-related chemokines (CXCL9, CXCL10). In addition, 9p arm-level loss and *JAK2-CD274* codeletion (at 9p24) were predictive markers of poor survival in recurrent HPV-negative HNSCC after anti–PD-1 therapy. As a consequence of the profound decrease in the chemokines CXCL9 and CXCL10, tumor-antigen cross-presentation and T cell priming and activation are most affected since these IFNγ-inducible chemokines are known to be secreted by dendritic cDC1 cells^[87,88]^. Further dissection of the 9p21 and 9p24 loci credits 9p21.4 loss as the key somatic alteration to shape the immune TME response. Conversely, 9p24.1 gain may act as a driver of immune activation and ICB response in HPV-negative HNSCC^[89]^. Together, 9p loss promotes T cell depletion and defective IFNγ-related pathways (CXCL9, JAK2 signaling), with enrichment of suppressive cells (Tregs, MDSCs)^[87,88,90]^. Hence, 9p loss has been proposed as a biomarker that could better predict the clinical benefit of ICB.

The proliferation and accumulation of oncometabolites in the TME give evidence for the metabolic state in cancer. This evidence is related to the requirement to maintain aerobic glycolysis, glutaminolysis, or one-carbon metabolism.

## CHANGES TO MAJOR METABOLIC PATHWAYS AND TREATMENT RESISTANCE IN HPV-NEGATIVE HNSCC

**Figure 3:**
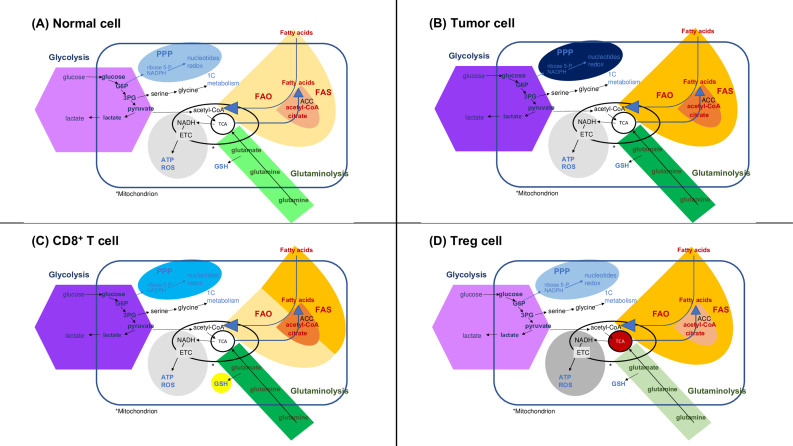
Metabolic pathways in (A) normal cells, (B) cancer cells, (C) CD8^+^ T cell, and (D) Treg cell . (A): Glycolysis, the conversion of glucose to pyruvate, and the pentose phosphate pathway (PPP), that generates ribose-5-phosphate and NADPH (required for nucleotide synthesis, redox balance, and fatty acid synthesis. Pyruvate can be converted to lactate and secreted or enter the tricarboxylic (TCA) acid cycle in the mitochondria. Fatty acids can undergo fatty acid oxidation and glutamine glutaminolysis, and enter the TCA. The TCA generates NADH and FADH that can enter the electron transport chain (ETC) and contribute to the synthesis of ATP and reactive oxygen species (ROS). Citrate from the TCA can enter the cytoplasm to participate in fatty acid synthesis (FAS). Glutamine metabolism can also synthesize glutathione (GSH); (B): cancer cells increase glycolysis, lactate production, PPP, FAO, FAS, and glutaminolysis. Cancer cells also maintain certain levels of the TCA cycle and oxidative phosphorylation (OXPHOS); (C): CD8^+^ T cells increase glycolysis, fatty acid uptake, FAS, glutamine uptake, glutaminolysis, and glutathione synthesis. The limited entry of pyruvate to the TCA favors the expression of IFNγ; (D): Tregs increase FAO, the TCA cycle, and OXPHOS; maintain PPP and glycolysis to obtain pyruvate, which feeds the increased flux of the TCA cycle and OXPHOS, while the conversion of pyruvate to lactate is restricted. Tregs limit FAS and glutamine metabolism. Increased fluxes are indicated by higher color tones.

As tumors grow, metabolism in the TME switches from oxidative phosphorylation to a glycolytic pathway favoring TME acidification. According to recent research^[91]^, metabolic plasticity in cancer cells has been demonstrated to improve antioxidant defenses and DNA repair activities, both of which can limit the efficacy of anticancer treatments [Figure 3]. In addition, the metabolic activity of cancer cells is highly plastic in response to a wide variety of environmental stresses, such as hypoxia and cytotoxic treatments, such as chemotherapy and RT. Finding new therapeutic targets for treating HNSCC could be the result of research into how ionizing radiation and other therapies affect the metabolism of cancer cells.

### Oxidative stress

Radiation induces oxidative stress that damages macromolecules, and when the excess of ROS that is generated after radiation exposure remains unrepaired, the oxidative stress can be lethal. Cancer cells with strong metabolic flexibility benefit from antioxidant effects and the DNA repair metabolites provided by metabolic rewiring. So, strong antioxidant defenses are crucial to minimizing radiation susceptibility. Tumor redox metabolism was discovered as a strong predictor for radiation sensitivity in TCGA patient tumors, including HNSCC^[92]^, showing a reliance on metabolic routes that accelerate the clearance of ROS and sustain antioxidant mechanisms as a driver of radiosensitivity. It is possible that metabolically focused therapies could modulate radiation sensitivity in this context. Numerous metabolic processes, such as glycolysis, the pentose-phosphate pathway (PPP), glutaminolysis, and one-carbon metabolism, play a role in antioxidant defenses and DNA repair. Metabolic reprogramming in cancer cells has been extensively reviewed in other publications^[91,93,94]^.

#### Glycolysis and the pentose phosphate pathway

One of the main nutrients that aid in the development and survival of cancer cells is glucose. Despite the fact that glycolysis is a highly inefficient process for producing ATP, rapidly proliferating tumor cells, which require large amounts of ATP molecules due to their unsustainable rate of replication, may use glycolytic intermediaries as precursors for the anabolic pathways and biomolecules necessary for cancer cells to survive^[95]^. The mechanisms of glucose metabolic reprogramming vary by cancer type and sometimes even within tumors of the same origin^[96]^.

Elevated levels of lactate and pyruvate in cancer cells drive the Warburg effect thanks to the overexpression and activation of glycolytic regulatory enzymes such as hexokinase (HK), phosphofructokinase (PFK) and pyruvate kinase (PK). In addition, the enhanced cellular transportation of metabolites through glucose transporters (GLUTs) maintains the Warburg effect. As a result of radiation, glucose metabolism is increased, which improves antioxidant activity and nucleic acid synthesis. The expression of GLUT1 has been associated with resistance to radiation in HNSCC^[97,98]^. Activation of glycolysis (via HK2) and HIF1 in HPV-negative HNSCC cells has been shown to induce radioresistance^[99]^. The activity of glycolytic intermediates that respond to oxidative stress increases after radiation. Namely, the activity of the PPP is mediated through the activation of the transcription factor nuclear factor erythroid 2‐related factor 2 (NRF2), a master regulator of enzymes involved in ROS detoxification and elimination, via inactivation of its negative regulator Kelch‐like ECH‐associated protein 1 (KEAP1), which facilitates the transcription of NRF2 target genes (including metabolic genes that drive the PPP) to generate NADPH that fuels the antioxidant systems. Preclinical models have identified NRF2 as predictive of radioresistance in oral cavity SCC^[100]^. Moreover, the PPP can be activated via ataxia telangiectasia mutated (ATM), a redox-sensitive kinase activated in response to radiation-induced DNA damage, by inducing the activity of glucose 6‐phosphate dehydrogenase (G6PDH).

#### Pyruvate and lactate

NAD+ is provided by the conversion of pyruvate to lactate in a highly glycolytic phenotype. Lactate production results in a requirement for lactate transport, both to prevent lactate accumulation and to provide a respiratory substrate. The temporal dynamics of the lactate-to-pyruvate ratio after radiation show a decrease shortly after radiation and increased levels over time, suggesting that redox modulation after radiation evolves over time^[91]^. Monocarboxylate transporters (MCTs) are the major players in the context of lactate exchange in tumor tissue. MCT1 strongly influences lactate levels and has been suggested as a potential biomarker of chemoradiation response in HNSCC, regardless of HPV status^[101]^. Further studies addressing the temporal dynamics of the altered lactate synthesis and transport following radiation are required for a better understanding of associated pathways and proposal of new targets^[91]^.

Lactate is used as a fuel^[102]^, but it also promotes tumor growth^[103]^. In addition, recent findings suggest that blocking the effects of intratumoral acidity while keeping CD8^+^ T cells’ lactate metabolism at a normal level can enhance antitumor immunity^[104]^. For T cell function specifically, lactate appears to represent a fundamental carbon source^[105]^ and further promotes the stemness of CD8^+^ T cells associated with the inhibition of histone deacetylase activity^[106]^. A fraction of highly glycolytic HNSCC stem cells is driven by epigenetic alterations that have been found. Antioxidant defense and nucleotide synthesis are both bolstered by a gene profile associated with glutathione (GSH) metabolism and stemness in this SIRT6 loss model^[107]^. SIRT6 is a member of the sirtuins, a family of negative regulators of HIF1-dependent glycolysis. There is mounting evidence that increased glycolysis is a hallmark of HNSCC.

#### Amino acids and one-carbon metabolism

Cancer cells require amino acids as a source of energy. Next to glucose, glutamine is the most important nutrient for cancer cells since it is used in the creation of proteins and nucleic acids. It is becoming increasingly clear that glutamine is essential for metabolic remodeling in cancer under oxidative stress. One main cause for the enhanced GSH production is glutaminolysis, which is a primary source for tricarboxylic acid cycle (TCA)-derived biosynthesis^[108]^. This makes glutamine very necessary for ROS scavenging and anabolic needs.

One of the limiting steps in glutaminolysis is the activity of glutaminases. An emerging strategy for interfering with cancer metabolism and tumor progression is targeting glutaminolysis by inhibiting glutaminases^[109]^. Studies imply that targeting a specific glutaminase metabolic route is unlikely to become a successful anticancer strategy due to the TME’s heterogeneity, the interconnected nature of cellular metabolism, and the plasticity of intracellular metabolic pathways. Having a better idea of which cells are using glutamine and in what pathways could help narrow down the possibilities^[110,111]^.

The increased need for glutamine in cancer cells is met by the solute carrier (SLC) family of membrane transporters, which includes the SLC1, SLC1-6, SLC1-7 and SLC1-38 families. Emerging evidence indicates that extracellular glutamine promotes ferroptosis, an iron-dependent cell death mechanism characterized by excessive generation of lipid peroxidation, which has been shown to induce cell death through ROS accumulation in cells. Of interest, it has been recently found that radiotherapy leads to lipid oxidation and ferroptosis via repression of SLC7A11^[112]^. It is noteworthy that lipid membrane composition regulates radiation sensitivity, indicating that lipid metabolism may be therapeutically addressed to enhance RT efficacy. Radiation resistance and decreased RT induction of ferroptosis and lipid peroxidation are related to high NRF2 and SLC7A11 expression^[113]^.

#### Nucleotide metabolism

The synthesis of nucleotides is highly required for DNA repair of double-strand breaks after radiation damage, especially in less radiosensitive cell types^[94]^. Consequently, an efficient DNA damage response (DDR) is linked to radiation resistance in cancer cells. Alkylating agents, such as cisplatin and lipid peroxidation resulting from ROS accumulation, can generate DNA adducts that also result in metabolic reprogramming through all major pathways (reviewed in ref.^[114]^). *De novo* synthesis of nucleotides, which affects DNA replication and repair, requires certain metabolites, such as glutamine and aspartate. The DDR’s activity is affected by cellular metabolism because of changes in substrate availability. The DDR is capable of regulating metabolic pathways that cause or protect against DNA damage, as well as those that rearrange chromatin and are necessary for DNA repair^[115]^.

Signaling via purine nucleotides and nucleosides, such as adenosine and adenosine 5^′^-triphosphate (ATP), is increased in HNSCC^[116]^. The balance between ATP (pro-inflammatory) and its catabolite, adenosine (anti-inflammatory), is tightly controlled in immune microenvironments at multiple levels. Adenosine signals in DCs upregulate IL-10, TGF-β, and arginase-2, promoting tumor growth. In TAMs, adenosine induces pro-tumor M2 macrophage polarization by reducing the expression of interleukin 2 (IL-2), tumor necrosis alpha (TNFα), and nitric oxide but upregulating arginase-1, IL-10, and vascular endothelial growth factor (VEGF)^[117]^. However, the balance is shifted to increased adenosine production in tumors, rendering a deeply immunosuppressed TME^[118]^. Moreover, ATP is rapidly converted into adenosine in the TME by the ectonucleotidases CD39 and CD73, which are particularly expressed in CD25^+^ or FoxP3^+^ Tregs. Hypoxia, via HIF1α, upregulates CD39 and CD73, further enhancing the adenosine pathway via adenosine receptor A2A (A2AR) in HNSCC^[119,120]^. Cancer cell death induced by RT releases ATP dose-dependently, which activates DCs and triggers an antitumor immune response. Conversion of ATP to adenosine can be generated directly by RT, via production of ROS, which activates TGF-β and promotes the M2 TAM phenotype^[121]^. Therapeutic targeting of A2AR, CD73, and TGF-β may shift the TME to a pro-ATP environment and reduce resistance to immunotherapy in the setting of RT. Purinergic signaling stands out as a particularly promising target for the development of novel anticancer agents, for the most part in combination with standard-of-care therapeutics or ICB^[122,123]^.

#### Fatty acids and lipids

Lipid metabolism is an area of cancer metabolic reprogramming that has received less attention. The need for metabolic intermediaries in macromolecule production is particularly great in cancer cells. In order to produce membrane-forming, energy-storing, signaling-molecule-generating and ATP-generating molecules, lipid metabolism is a crucial route. Alterations in lipid availability are associated with altered cancer cell motility, angiogenesis development, metabolic symbiosis, immune surveillance evasion, and treatment resistance. The reprogramming of fatty acid (FA) metabolism in tumor tissues has gained considerable interest as a potential cancer treatment target^[93]^. This suggests that FAs may be another environmental resource that CD8^+^ Teff cells need to compete with tumor cells in the TME^[124]^, given that CD8^+^ Teff cells also take up FAs at high rates.

The first stage in FA metabolism is for FA transporters (CD36/FAT, FABPpm, and FATPs) to bring FAs into the cell. In order to power keratogenesis and the TCA cycle, mitochondria convert imported FAs into fatty acyl-CoA before transporting it for oxidation. Lipogenesis enzyme overexpression, FA trafficking, and FA oxidation (FAO) are among the ways in which FA metabolism can be influenced. Key regulators of lipogenesis include stearoyl-CoA desaturase 1 (SCD1), fatty acid synthase (FASN), acetyl-CoA carboxylase (ACC), and the sterol regulatory element-binding proteins (SREBPs). There is a class of proteins called FA binding proteins (FABPs) that play a role in the transport of FAs within cells. Cancer cells rely heavily on FAO reprogramming. FAO generates cytosolic NADPH to assist biosynthesis and can produce twice as much ATP as carbohydrates.

Studies on FAs in patients with HNSCC are limited. Abnormal elevation of the FA transporter CD36 in oral HNSCC promotes tumor metastasis. In contrast, its inhibition leads to complete remission or elimination of lymph node and lung metastases in *in vivo* models^[125]^. In fact, acidosis-induced TGF-β2 activation stimulates both partial epithelial-to-mesenchymal transition and FA uptake via CD36 that are stored in lipid droplets (LD). LDs represent energy stores for cancer invasion and dissemination^[126]^. The lipogenesis enzyme FASN was shown to promote radiation resistance in preclinical HNSCC models^[98]^.

There is a correlation between radiation, lipid redox, and ferroptosis that has recently been established in the literature. Liposomes undergo peroxidation and lipid fragmentation after radiation, while radiolysis generates oxygen radicals that cause lipid peroxidation of polyunsaturated FA (PUFA), in a radiation dose-dependent manner^[127]^. The excess of lipid oxidation leads to ferroptosis. Ferroptosis is a form of cell death induced by iron-dependent lipid peroxidation. Ferroptosis induction is dictated by the proportion of PUFA in the lipid membrane^[128]^. Thus, targeting lipid metabolism may accentuate the effect of radiation. Upregulation of SLC family members that regulate cystine import (critical for GSH biosynthesis and maintenance of the antioxidant pool within the cell) has been related to acquired RT resistance. The suppression of SLC7A11 by RT-activated ATM results in decreased cystine absorption, enhanced tumor lipid oxidation and ferroptosis, and improved tumor control^[112]^. Additionally, low levels of RT-induced ferroptosis and lipid oxidation are associated with high levels of NRF2 and SLC7A11 expression, which is linked to resistance to RT^[113]^. In HNSCC, preclinical models suggest that NRF2 inhibition could restore cisplatin sensitivity^[129]^. In another model, the inhibition of the glutamine transporter SLC1A5, expressed at high levels in the CD44 variant‐cancer stem-like cells (SLC1A5^+^/CD44v^high^), triggered oxidative damage^[130]^. Taken together, these findings show that cystine transporters represent an original and targetable mechanism to improve RT efficiency.

It has been hypothesized that PMN-MDSCs in the TME of HNSCC use ferroptosis as an immunosuppressive mechanism. Even though ferroptosis reduces PMN-MDSC numbers in the TME, it inhibits T cell function by increasing the release of immunosuppressive chemicals. It has been postulated that ferroptotic PMN-MDSCs exert suppression by soluble substances, possibly including prostaglandin E2, and is favored under hypoxic settings, in contrast to their main mechanism in peripheral lymphoid organs, which is direct interaction between PMN-MDSCs and T cells^[131]^. Additional work is required to understand how to integrate ferroptosis into the clinical setting.

### Role of hypoxia

Rapid tumor cell proliferation in the TME creates low-oxygen regions with uneven concentrations of oxygen, leading to hypoxic stress. Extreme low oxygen levels stimulate a tolerant TME that allows evasion of immune surveillance^[132]^. In particular, the hypoxic TME encourages the formation and production of immunosuppressive cells and secretion of substances (e.g., VEGF and TGF-β), and upregulation of immune checkpoint molecules on cancer cells to limit the capacity of effector immune cells to eradicate cancer cells. Under hypoxic conditions, the tumor ECM stimulates collagen synthesis and maturation, thereby generating a highly dense ECM^[133]^. The stabilization of HIF-signaling by hypoxic cells alters cellular redox and increases the use of glycolysis and alternate metabolizing pathways, which in turn increases resistance in the surrounding cells^[134]^.

In contrast, hypoxic signaling may trigger angiogenesis, which will cause reoxygenation of tumor tissue, and enhanced radiosensitivity. In spite of this, there has been evidence that radioresistance persists after reoxygenation of hypoxic cells caused by G1 arrest/quiescence. These findings may suggest that tumor cells in chronic hypoxic regions, which are often located near necrotic zones due to an inadequate oxygen supply^[135]^, acquire a quiescent state^[136]^ and that reoxygenation alone is not sufficient to re-sensitize previously hypoxic cells. Notably, in cases of HPV-positive HNSCC, this condition disappears entirely. This would suggest that hypofractionation may maximize cancer cell death, while the interfraction interval should be tuned to provide optimal re-entry of previously hypoxic cells into the cell cycle^[19]^.

It has been shown that HNSCC patients with persistent tumor-associated hypoxia during chemoradiation had a worse outcome^[137]^ linked to high levels of tumor-infiltrating lymphocytes^[138]^, highlighting the clinical relevance of persistent hypoxia in HNSCC.

## BRIDGING THE GAP

The local TME of the tumor and T cell priming in the lymph nodes greatly define the potency of the cytotoxic immune response^[139]^. DCs play an essential role in CD8^+^ T cell differentiation and antitumor activity^[140]^. Preclinical research to exploit these traits^[25,74,141]^ has been clinically translated recently, offering new strategic opportunities in HPV-negative HNSCC. In addition, considering the more active role of tumor-draining lymph nodes merits consideration.

### Innovative preclinical models to evaluate the TME in HNSCC

Apart from animal models, other tools have been incorporated to account for the effect of immune infiltration in the TME. In the context of personalizing treatment options, major efforts are being directed towards the development of reliable three-dimensional experimental models to study the microenvironment and treatment-resistance mechanisms.

Amongst them, organotypic co-culture models to culture fresh HNSCC tumor explants allow cultivation of cancer slices for up to 21 days in their original tumor microenvironment and investigation of the clonal expansion of resistant cancer cells upon treatment^[142]^. A lymphatic organotypic microfluidic model has been proposed to study lymphangiogenesis, consisting of a lymphatic vessel surrounded by primary tumor-derived fibroblasts, which offers the potential to evaluate HNSCC metastasis to lymph nodes via lymphatic vessels accounting for the heterogeneity of the individual TME^[143]^. An alternative system is a biomimetic collagen-based scaffold, which has been used to study the impact on the phenotype and genotype of oropharyngeal cancer cells. The 3D architecture in this model enables the study of the induction of migration properties and of the expression of epithelial-mesenchymal transition markers ^[144]^.

One emerging field uses microfluidic platforms to study the TME and how immune cells and tumor cells interact with it. Organs-On-Chip are innovative tools that have boosted the research on cell-cell interactions and migratory behaviors. In the oncoimmunology field, these platforms are called OncoImmuno chips, and in their most ambitious objective aspire to become a Human-On-Chip (HOC, a multicellular chip module that contains all the key cellular components and extracellular factors derived from a specific human donor’s immune cells)^[145]^. In this regard, a HOC recapitulating the systemic metastatic spread has been proposed, composed of different modules that in the near future could be connected to generate a working metastasis HOC^[146]^.

### Clinical translation

Because immunological dysfunction can develop over time and while treatment is being received^[147]^, the optimal timing of immunotherapeutic intervention, such as neoadjuvant therapy^[148]^, window of opportunity trials^[149]^ for surgically resectable HNSCC, concurrently with standard definitive treatment, or as adjuvant or salvage therapy, has yet to be determined.

Patients with HPV-negative HNSCC who participated in a recent phase I/Ib trial and were treated with neoadjuvant hypofractionated stereotactic body radiotherapy (SBRT) with a single dose of durvalumab had an overall survival of 80.1% (95% CI, 62%-100%), locoregional control/progression-free survival of 75.8% (95% CI, 57.5%-99.8%), and major/complete pathological response of 75% (95% CI, 51.6%-100%)^[150]^. It has been demonstrated that combining immunotherapies with RT can boost the infiltration of immune cells into the TME, and immunological priming is the intended outcome of this research. It has been demonstrated that SBRT is able to increase antitumor immune function by increasing the abundance of T cells in the TME and activating those T cells. A single dosage of the neoadjuvant durvalumab is administered to patients anywhere from three to six weeks prior to the scheduled standard operation. This dose was provided concurrently with neoadjuvant SBRT to regions that exhibited clear signs of disease. This was escalated using a 3 + 3 model. Initially, adjuvant therapy based on pathology was used, but in the expansion cohort, none of the patients who achieved pathological major or complete response received adjuvant treatment^[74]^. All of the patients received adjuvant durvalumab between 6 and 12 weeks after their surgeries, with a maximum of 6 doses per patient. It was concluded that a total of 24 Gy, split up into three doses, is the maximum dose of SBRT that can be safely given. The maximal pathological response was not seen until at least 5 weeks after radioimmunotherapy was finished. This likely indicates the amount of time required to create systemic immunological memory. Specifically, a biological correlation research study found that patients who had a positive response to treatment had higher levels of Teff cells, lower levels of immunosuppressive cells, and improved antigen presentation. The changes that occurred in the draining lymph nodes (DLNs) of responders provided evidence for enhanced antigen presentation and T cell priming in the DLNs. This was demonstrated by an indication of a shorter distance between DCs and T cells. It has been observed that when CD8^+^ T cells were located closer to cancer cells in the TME, outcomes were much better than using the overall number of CD8^+^ T cells^[151]^. The findings of this research suggest that the two patterns are related.

In a different trial, a phase Ib/IIa study testing neoadjuvant combined checkpoint blockade prior to surgery in HNSCC, two doses of nivolumab plus one dose of ipilimumab were administered 4 weeks before conventional surgery, and resulted in 35% of major pathological response^[152]^. Notably, the response was discordant between primary HNSCC and its lymph node metastases. As it might be a matter of time to surgery, other potential explanations might apply.

### A role for tumor-draining lymph nodes in immunotherapy

A lymph node has been found to have a lipid-rich milieu that tumor cells may preferentially utilize as an energy source^[153]^. Moreover, new evidence suggests that FAO can aid in the settling of circulating tumor cells (CTCs) in lymph nodes^[154]^. This lymph node pre-metastatic niche (PMN) requires lymphangiogenesis, recruitment of immunosuppressive cells, upregulation of chemokines and cytokines and vascular remodeling^[155]^. Proliferation and differentiation of lymphatic endothelial cells cause lymphangiogenesis, which has been demonstrated to be regulated by FAO^[156]^. Tregs, MDSCs, TAMs and pDCs within the PMN potentially suppress antitumor immune responses. However, Teff cells, being at a disadvantage in competing for glucose with tumor cells, redirect their metabolism towards FAs. Furthermore, Tregs may augment FAs, thus influencing effector cell development. FAO dominates in M2 TAMs, favoring interleukin 1 beta (IL-1β) secretion and tumor cell migration^[157]^. MDSCs activate FAO enhancing the ability to suppress T cell function. Recent studies show that stromal and immune cells in the PMN secrete substances in concert with FAO, including TGF-β and vascular endothelial growth factor-C (VEGF-C)^[158]^. Vascular remodeling, also known as tumor cell-induced microvascular expansion, may increase angiogenesis and vascular permeability to promote metastasis^[159]^. As a result of an increase in HIF1 and hypoxia-induced proangiogenic factors, CTCs from the vasculature and lymph vessels may reach the PMN and recruit more tumor cells. Modifications to the microenvironment of the lymph node, such as lymphangiogenesis, an increase in the expression of immunosuppressive cytokines, and an increase in the number of immunosuppressive cells, make it possible for tumor cells to thrive and remain dormant until stimuli drive their progression into a micrometastasis.

The potential role of the lymphatic system in driving immune evasion and metastasis has become highly relevant in cancer research^[26]^. The state of the TME may become functionally altered depending on lymphatic activation states. In support of the hypothesis that lymph node metastasis is a functional driver of disease, a recent publication finds that metastatic lymph nodes display Treg expansion that generates tolerance to tumor cells and enhanced metastatic potential^[160]^. The effect of increasing tumor burden remains to be determined. There is evidence of a progressive loss of T cell proliferation accompanied by the proliferation of Tregs^[27]^. Whether this hypothetical setting can influence the established role of complete lymphadenectomy, which has shown limited survival impact, or provide an opportunity for neoadjuvant immunotherapy to target and remodel regional lymph nodes, remains to be seen. An improved understanding of lymphatic adaptation in tumor progression and response to immunotherapy could lead to more effective treatment strategies.

## CONCLUDING REMARKS

**Figure 4:**
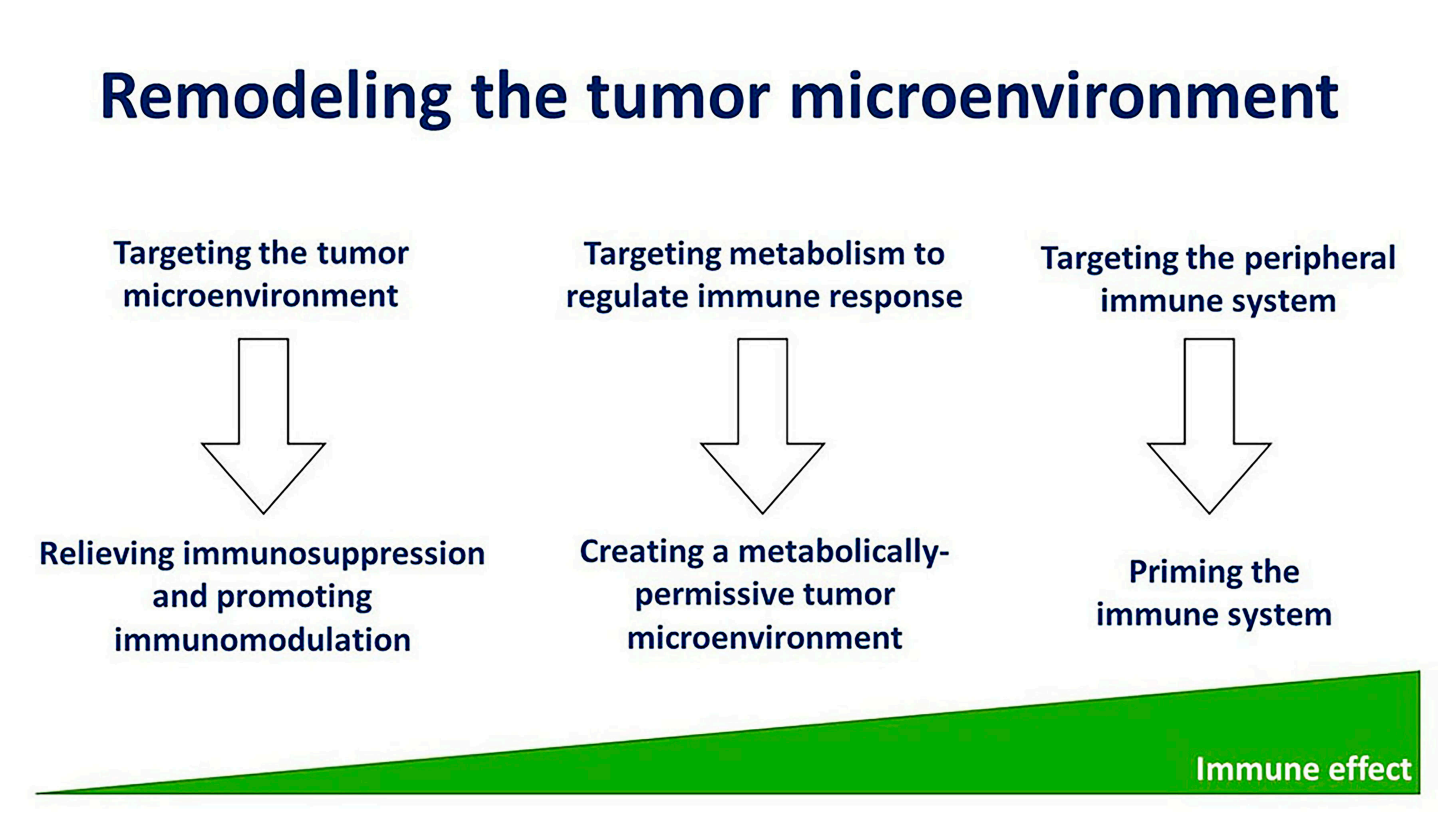
Immunomodulation, creating a metabolically permissive microenvironment, and priming the immune system to remodel the tumor microenvironment and elicit a strong immune response.

The multiplicity of immune evasion mechanisms that immune-cold tumors can adopt is a major barrier to benefit from immune therapy strategies. The impact of immunotherapy in HPV-negative HNSCC has been rather low, indicating that a better understanding of the immune escape mechanisms in these tumors can definitively enhance their immunogenicity and treatment response. This review proposes that modulation of the TME to relieve immunosuppression, creation of a metabolically permissive TME, and priming the immune system are promising strategies to remodel the TME to overcome treatment resistance in HPV-negative HNSCC [Figure 4]. However, the concepts that have been discussed have yet to be implemented in the clinic, reflecting the preliminary stage of this information. Nonetheless, a few salient points can be summarized.

Evidence that an efficient antigen presentation machinery elicits a potent immune response in HNSCC has been studied in two phase I-I/II trials^[150,152]^ with strong accompanying correlative studies. Major or complete pathological responses of 75% were shown after neoadjuvant SBRT to gross disease and a single dose of durvalumab, followed by adjuvant durvalumab of up to 6 doses^[150]^. The biological correlates indicated that response was related to higher levels of Teff cells, lower levels of immunosuppressive cells, and improved antigen presentation and T cell priming in the draining lymph nodes. Of note, when DCs and T cells were closer in the draining nodes, and CD8^+^ T cells and tumor cells were closer in the TME, outcomes were better^[151]^. In preclinical models, Treg depletion or reprogramming strategies in combination with radiation have proven to activate DCs^[72-74]^. A central tenet of this strategy is the use of SBRT only on gross disease, which optimizes the presence of neoantigens and allows a better coordinated immunologic response in the draining lymph nodes. Additional data confirmation and a refined consideration of the different contributions of the immunosuppressive cells that compose the heterogeneous TME will further guide the strategic scope of this treatment approach.

Robust data exist that indicates that the functionality of the IFNγ-inducible chemokines CXCL9 and CXCL10, essential for the recruitment of CD8^+^ T cells and NK cells, favors T cell trafficking and infiltration. Multiomic analyses of HPV-negative HNSCC cohorts have identified that losses at genomic regions on the chromosome 9p containing IFNγ-pathway genes result in CD8^+^ T cell depletion and CXCL9/CXCL10 suppression, and predict immune-cold, ICB-resistant tumors^[87-89]^. Pan-cancer studies suggest that this mechanism is prominent in HNSCC and pancreatic cancer^[86]^, and has become a biomarker that synergizes with PD-L1/TMB for patient stratification. Further genomic/non-genomic dissection of these alterations can provide new strategies to target these tumors.

Studies evaluating the metabolic alterations that occur in the TME have uncovered potential treatment targets, like the transcription factor NRF2 which supports the PPP^[100]^. Preliminary data suggest that the amino acid cysteine may play an important role in mediating CD8^+^ T cell-induced ferroptosis^[161]^, a recently identified form of regulated cell death. Ferroptosis, the mechanism by which abnormal intratumoral lipid metabolism induces cell death, has attracted great attention as a potential novel target in oncology^[162]^. The discovery that radiotherapy induces ferroptosis in cancer cells as a result of an ATM-mediated downregulation of SLC7A11, and that this effect is enhanced in combination with ICB in animal models, has evolved into an ongoing active area of investigation^[112]^.

In conclusion, while additional research is needed, confidence exists that well-designed preclinical and clinical studies to assess neoadjuvant SBRT schedules in combination with immunotherapy, the clinical applicability of 9p loss, and the role of ferroptosis in cancer will elucidate which patient subgroups benefit the most. A better definition of contributing factors of an immunogenic microenvironment constitutes a significant step forward, which could be further exploited by incorporating emerging factors like genomics or the influence of the microbiome^[163]^.

## DECLARATIONS

### Copyright

© The Author(s) 2023.
